# Rapid Screening for α-Glucosidase Inhibitors from *Gymnema sylvestre* by Affinity Ultrafiltration–HPLC-MS

**DOI:** 10.3389/fphar.2017.00228

**Published:** 2017-04-27

**Authors:** Guilin Chen, Mingquan Guo

**Affiliations:** ^1^Key Laboratory of Plant Germplasm Enhancement and Specialty Agriculture, Wuhan Botanical Garden, Chinese Academy of SciencesWuhan, China; ^2^Graduate University of Chinese Academy of SciencesBeijing, China; ^3^Sino-Africa Joint Research Center, Chinese Academy of SciencesWuhan, China

**Keywords:** *Gymnema sylvestre*, gymnemic acid, α-glucosidase, UF-HPLC-MS, high-throughput screening

## Abstract

*Gymnema sylvestre* R. Br. (Asclepiadaceae) has been known to posses potential anti-diabetic activity, and the gymnemic acids were reported as the main bioactive components in this plant species. However, the specific components responsible for the hypoglycemic effect still remain unknown. In the present study, the *in vitro* study revealed that the extract of *G. sylvestre* exhibited significant inhibitory activity against α-glucosidase with IC_50_ at 68.70 ± 1.22 μg/mL compared to acarbose (positive control) at 59.03 ± 2.30 μg/mL, which further indicated the potential anti-diabetic activity. To this end, a method based on affinity ultrafiltration coupled with liquid chromatography mass spectrometry (UF-HPLC-MS) was established to rapidly screen and identify the α-glucosidase inhibitors from *G. sylvestre*. In this way, 9 compounds with higher enrichment factors (EFs) were identified according to their MS/MS spectra. Finally, the structure-activity relationships revealed that glycosylation could decrease the potential antisweet activity of sapogenins, and other components except gymnemic acids in *G. sylvestre* could also be good α-glucosidase inhibitors due to their synergistic effects. Taken together, the proposed method combing α-glucosidase and UF-HPLC-MS presents high efficiency for rapidly screening and identifying potential inhibitors of α-glucosidase from complex natural products, and could be further explored as a valuable high-throughput screening (HTS) platform in the early anti-diabetic drug discovery stage.

## Introduction

Characterized by high blood glucose levels, diabetes mellitus (DM) has become one of the most serious chronic endocrine metabolic dysfunction. According to the WHO, 90% of the 382 million DM patients worldwide were type 2 diabetes mellitus (T2DM) in 2013, which still shows tendency of growing. With the increase of age, many severe long-term complications, e.g., diabetic retinopathy, kidney failure, cognitive decline, and diabetic neuropathy, will badly affect the T2DM patient's physical and mental health. Furthermore, repeated postprandial hyperglycemia may facilitate the development of the above serious adverse effects, and, in extreme cases, the risk of mortality. In clinical trials, those complications could be delayed or prevented by the intensive postprandial hyperglycemia control (Li et al., 2015; Liu et al., 2016; Zhang et al., 2016). To this end, postprandial blood glucose, especially in non-insulin-dependent T2DM patients, has attracted growing attentions as a potential therapeutic target.

As the major part of the daily diet, carbohydrates were firstly hydrolyzed into monosaccharides, and then absorbed via the small intestine. α-Glucosidase, an enzyme secreted in the small intestinal chorionic epithelium, has been proved to catalyze the cleavage of oligosaccharides and disaccharides into monosaccharides during the final step in the digestions of the carbohydrates. Studies showed that α-glucosidase inhibitors can delay or reduce the digestion and absorption of carbohydrates, and thus decrease the postprandial blood glucose level (Khan et al., 2014; Zhou et al., 2015). α-Glucosidase inhibitors, which were classified as the oral hypoglycemic agents of third category (e.g., acarbose and voglibose) and exhibited significant inhibition on postprandial blood glucose in the T2DM patients, have been recommended as a first line therapy by IDF (International Diabetes Federation) and AACE (American Association of Clinical Endocrinologists). In consideration of the outstanding health promoting effects, natural products have been considered as important drug source and served for the medicinal pharmaceuticals or herbal remedies for thousands of years worldwide. Therefore, natural α-glucosidase inhibitors with no or less side reactions have gradually attracted broad interests for the treatment of T2DM around the world.

*Gymnema sylvestre* R. Br. (*G. sylvestre*), a woody climbing plant of the Asclepiadaceae family, is largely distributed in southwestern China and India. As a traditional folk medicine, the leaves of this plant have been known as “Gurmar” for their obvious sweet taste inhibiting property in India, and commonly consumed for the treatment of diabetes mellitus, obesity, eye complaints, asthma in food additives, and snake bite. Reports showed that the crude saponin fraction from the leaf extracts exhibited antihyperglycemic effect through decreasing the glucose absorption in the small intestine and then suppressing the postprandial blood glucose level in human and rats (Suttisri et al., 1995; Persaud et al., 1999; Ye et al., 2000). Phytochemical investigations further indicated that the bioactive components responsible for the the hypoglycemic activity of this medicinal foodstuff mainly consisted of triterpene saponins, namely gymnemic acids, and their analogs (Sahu et al., 1996; Fabio et al., 2014). And the flavones, anthraquinones, lupeol, *d*-quercitol, β-amyrin related glycosides, resins, alkaloids, and stigmasterol constitute to other chemical constituents of this plant (Praveen et al., 2014). Up to now, a number of compounds, such as gymnemic acids, deacyl gymnemic acids, gymnemagnenins, and gymnestrogenins, have been isolated and purified from *G. sylvestre* (Ye et al., 2001; Liu et al., 2004; Daisy et al., 2009). However, previous investigations on the evaluations of anti-diabetic effects mainly focused on either the saponin riched fraction or some pure compounds, the specific bioactive components responsible for this health promoting effect in this plant remain no definite conclusions.

The conventional approaches for screening the bioactive ingredients from natural products require bioassay-guided repeated column chromatography isolations, which are labor-intensive, time-consuming and low efficiency, and sometimes lead to the incidences of false positives/negatives with relatively high risk of failure by reason of irreversibly adsorption, decomposition, dilution effects (Zhang et al., 2013; Xiao et al., 2015; Zhou et al., 2015). Derived from the urgent needs of high-throughput screening of bioactive compounds in recent years, a combinational method of bio-affinity ultrafiltration and high performance liquid chromatography coupled with electrospray mass spectrometry (HPLC-ESI-MS/MS) has been proposed. In the process, the bio-affinity ultrafiltration separates the ligand-receptor complexes from the unbound compounds, and the ligands released from the complexes could be readily identified and subsequently quantified by HPLC-MS/MS analysis. Therefore, the ultrafiltration coupled with HPLC-MS (UF-HPLC-MS) method could not only identify numerous interesting and/or novel compounds without tedious prior isolations, but also illustrate the potential biological mechanisms by providing pivotal insights into bio-molecular structures and ligands binding properties with high specificity and sensitivity, which is very powerful for the rapidly screening and identification of potential ligands in natural products at the early drug discovery stage (Katoch et al., 2012; Qin et al., 2015; Chen et al., 2016). A number of researches have referred to this method to screen and identify ligands from complex natural mixtures (Zhu et al., 2013; Li et al., 2015; Qin et al., 2015; Tao et al., 2015; Chen et al., 2016). However, to our best knowledge, UF-HPLC-MS method has not been applied to screen and identify α-glucosidase inhibitors from *G. sylvestre*, not to say its metabolites. Inspired by the above applications and medicinal exploitations of *G. sylvestre*, we selected α-glucosidase as the drug target to establish UF-HPLC-MS assay to rapidly screen and identify major α-glucosidase ligands from *G. sylvestre*. To some extent, our present work offers a powerful tool for discovering bioactive components from complex natural products.

## Materials and methods

### Materials, chemicals, and reagents

The leaves of *G. sylvestre* (originated from Jiangmen city, Guangdong province) were purchased from Shiyuan pharmaceutical Co., Ltd. (Hebei, China), and then authenticated by our taxonomist, professor Guangwan Hu. A voucher specimen (No. 0033) was deposited in herbarium of the Key Laboratory. The air-dried samples were ground with a blender, packed in polyethylene bags, and then stored in the refrigerator at 4°C until use.

α-Glucosidase powder were obtained from Sigma-Aldrich (Missouri, USA). *p*-nitrophenyl α-D-glucopyranoside (*p*NPG) and acarbose were bought from Uteam-BIOTECH (Shanghai, China) and Yuanye Bio-Technology Co., Ltd (Shanghai, China), respectively. HPLC-grade acetic acid and acetonitrile (ACN) were purchased from TEDIA Company Inc. (Fairfield, Ohio, USA). D101 macroporous resin was obtained from an industrial chemical company affiliated to Nan Kai University (Tianjin, China). Centrifugal ultrafiltration filters (YM-30, 30 kDa) were provided by Millipore Co. Ltd (Bedford, MA, USA). Water for HPLC and LC-MS/MS was prepared with EPED (Nanjing Yeap Esselte Technology Development Co., Nanjing, China). All other chemicals and solvents were of analytical grade.

### Preparation of the extract of *G. sylvestre*

The raw powder of *G. sylvestre* (100.0 g) was accurately weighted and then extracted three times in an ultrasonic bath with 60% aqueous ethanol for 30 min at room temperature. After filtration with quantitative filter papers, the combined filtrates were concentrated by rotary vaporization under reduced pressure at 40°C to afford the syrup extract. The crude extract was redissolved in water and then subjected to liquid-liquid fractionation with petroleum ether (PE, b.p. 60–90°C), and n-butanol, successively. Then, the n-butanol fraction was eluted on a D101 macroporous resin column with distilled water to nearly colorless, and 60% aqueous ethanol stepwise. Finally, the 60% aqueous ethanol elution was lyophilized in a freeze dryer to dryness, and the residue (tested sample) was obtained for the subsequent analysis. Dimethyl sulfoxide (DMSO) stock solutions of *G. sylvestre* were prepared before use.

### α-glucosidase inhibitory assay

The α-glucosidase inhibitory assay was performed according to the method reported previously with some modifications (Zhang et al., 2016). In short, 100 μL of diluted sample and 200 μL of α-glucosidase solution (pH 6.8, 1.0 U/mL, in 10 mM phosphate buffer) were incubated at 37°C for 5 min. Then, 100 μL of 5 mM *p*NPG solution (pH 6.8, in 10 mM phosphate buffer) were added to initiate the reaction. After incubation at 37°C for 10 min, 40 μL of 0.1 M sodium carbonate (Na_2_CO_3_) solution were added to stop the reaction. Finally, the absorbance at 405 nm was recorded. The inhibition rate (%) was calculated as follows:

(1) $$\begin{array}{l} \text{Inhibition rate }\left(\%\right)=\frac{\text{Ac}-\text{As}}{\text{Ac}}\;\times \;100\% \end{array}$$

where Ac and As were the optical density (OD) values of control (without sample) and tested sample, respectively. Acarbose was used as the positive control. Each sample was tested in triplicate. The IC_50_ values of samples (the concentration resulting in 50% inhibition of α-glucosidase) were calculated by non-linear regression analysis (SigmaPlot, 12.5) and expressed as means ± *SD* (standard deviation).

### Screening potential α-glucosidase inhibitors with ultrafiltration

The present ultrafiltration screening procedure was performed with the ultrafiltration device, following the previous report on small modifications (Chen et al., 2016). The tested *G. sylvestre* sample and α-glucosidase were dissolved in 10 mM phosphate buffer (pH 6.8). Briefly, 100 μL of tested sample solution (2.0 mg/mL) was incubated with 200 μL of α-glucosidase (10 U/mL) for 30 min at 37°C. Meanwhile, inactivated α-glucosidase solution (boiled for 10 min in water bath) was used as the negative control in a similar manner. After incubation, the incubation solutions were ultrafiltrated through a 30 kDa molecule weight cut-off ultrafiltration membrane (YM-30) and centrifuged at 10,000 rpm to separate the unbound constituents from the α-glucosidase-ligand complexes for 10 min at room temperature. Then the filtrates were washed three times with 200 μL of 10 mM phosphate buffer (pH 6.8) by centrifugation to remove the unbound components. After that, the ligands showing specific binding to α-glucosidase were released from the complexes by incubation with 50% aqueous ACN for 10 min, and then centrifuged at 10,000 rpm for 10 min at room temperature, which was repeated twice. The combined filtrates were dried by lyophilization with a centrifugal evaporator. Finally, the residues were redissolved in 50 μL of 50% aqueous ACN and directly analyzed using the HPLC-ESI-MS/MS system.

### HPLC-MS/MS analysis

Experiments for the chromatographic separation were performed using a Thermo Accela 600 HPLC system (Thermo Fisher Scientific, San Jose, CA, USA). The HPLC system was equipped with a quaternary pump, an on-line vacuum degasser, an autosampler and a thermostatic column compartment, which were connected in line to a UV detector (2998). A 10 μL aliquot of sample solution was injected into a Sunfire™ C18 column (4.6 × 150 mm, 3.5 μm; Waters Symmetry, MA, USA). The mobile phase consisted of 0.1% formic acid-H_2_O (A) and 0.1% formic acid-ACN (B), and the linear elution procedures performed as follows: 0–2 min, 15% (B); 2–20 min, 15–55% (B); 20–25 min, 55–70% (B); 25–40 min, 70–95% (B); 40–45 min, 95–15% (B). The column temperature was set at 30°C with a flow rate of 0.5 mL/min, and the on-line spectra were obtained at the wavelength of 215 nm.

For the ESI-MS/MS analysis, a TSQ Quantum Access MAX mass spectrometer equipped with an electrospray ionization (ESI) interface was operated in the negative ion mode. The source voltage and desolvation temperature of the ionization conditions were set at 3.0 kV and 350°C, respectively. The capillary temperature was 250°C. The pressure of sheath gas (N_2_) and axu gas (He) were 40 psi and 10 psi, respectively. The collision energy ranged from 30 to 45 eV according to the mass of the precursor ion. The scan range for the mass spectra of *m/z* data was from 150 to 1,500 in the full-scan mode, and all the final data and analysis were acquired with the Thermo Xcalibur ChemStation (Thermo Fisher Scientific).

## Results

### α-glucosidase inhibitory activity of the *G. sylvestre*

**Figure 1:**
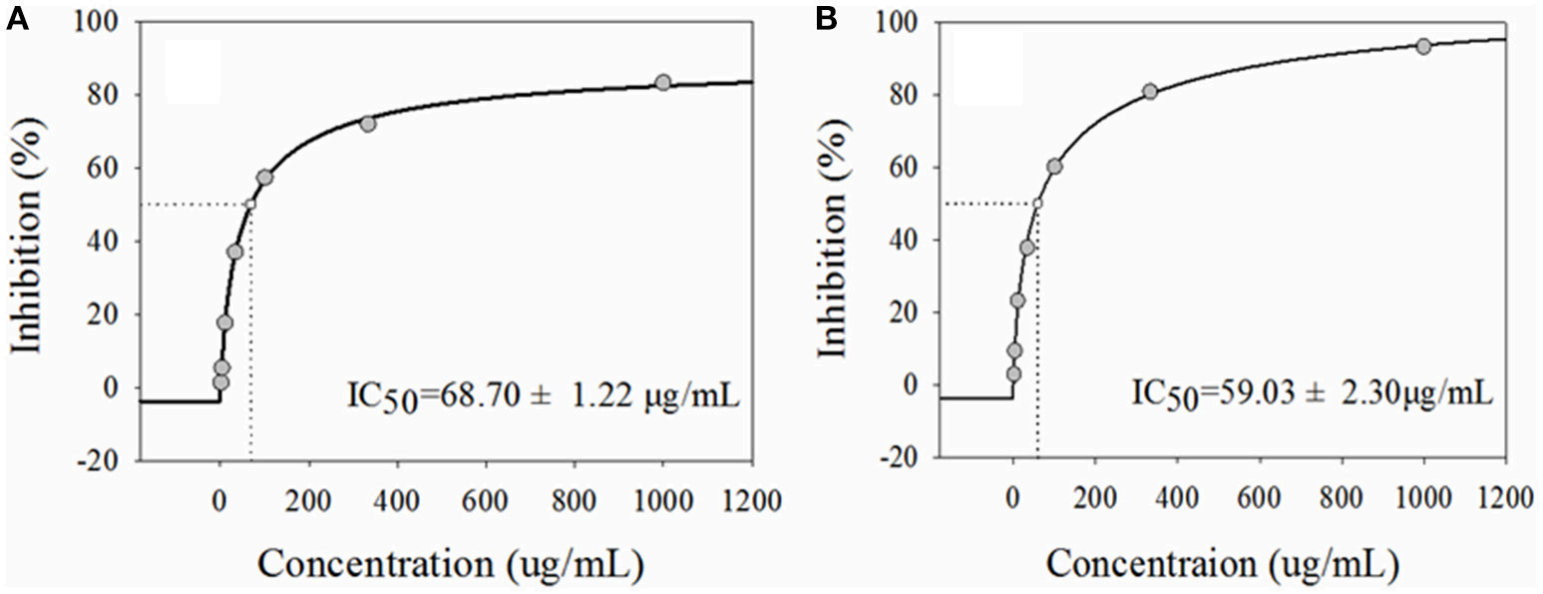
**The half-maximal inhibitory concentrations (IC_**50**_) of the extract from ***G. sylvestre*** (A)** and acarbose **(B)** on α-glucosidase *in vitro*. They exhibited comparable dose-dependent manners with the IC_50_ at 68.70 ± 1.22 and 68.70 ± 1.22 μg/mL, respectively.

The system solvent extraction has been commonly applied to partition complex natural extracts. In the method, 60% aqueous ethanol extract of *G. sylvestre* was fractionated by PE (to remove pigments) and n-butanol (to extract total saponins), successively. Then, those saponins were further enriched and purified with D101 macroporous adsorptive resins. After that, the tested samples of *G. sylvestre* at the indicated concentrations of 1.00–1000.0 μg/mL were performed to assess the inhibitory effects on α-glucosidase *in vitro*. Acarbose, a first line drug of reversible α-glucosidase inhibitor, was applied as the positive control. As shown in Figure 1, the extract of *G. sylvestre* displayed remarkable inhibitory activity against α-glucosidase with IC_50_ at 68.70 ± 1.22 μg/mL compared with the acarbose at 59.03 ± 2.30 μg/mL, which confirmed the potential anti-diabetic effect and indicated that the extract of *G. sylvestre* was abundant with α-glucosidase inhibitors. As a consequence, it would be valuable to rapidly screen and identify these active components in this herbal plant.

### Ultrafiltration analysis of the potential α-glucosidase ligands from the *G. sylvestre*

**Figure 2:**
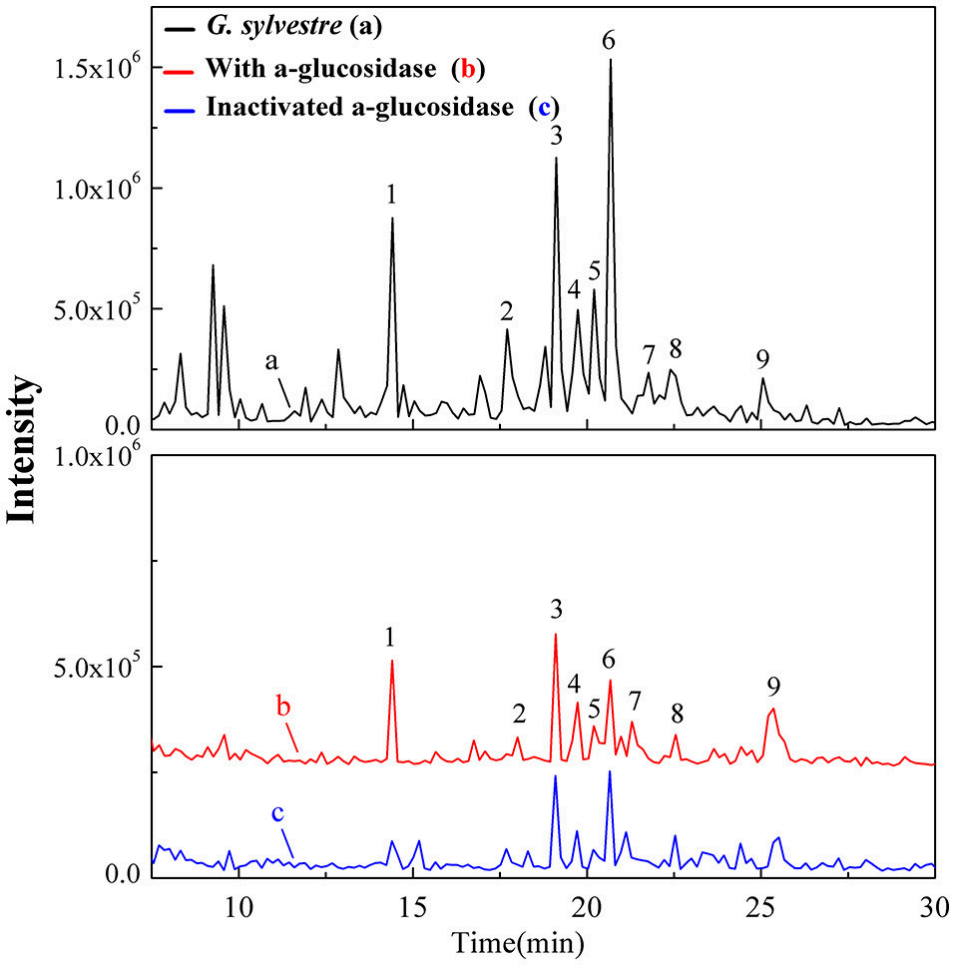
**The total ion chromatograms for the UF-HPLC-MS screening of the chemical constituents in the ***G. sylvestre*****. The black solid line (a) represents HPLC profiles of the extract of *G. sylvestre* without ultrafiltration; and the red line (b) and blue line (c) represent HPLC profiles of the *G. sylvestre* with activated and inactivated Top I, respectively.

The ultrafiltration-HPLC method could be applied to rapidly screen and identify bioactive ingredients from complex natural products without sample preparation. After incubation with α-glucosidase and affinity ultrafiltration, the bound ligands in *G. sylvestre* were released and then analyzed by HPLC-MS. Figure 2 shows the ultrafiltration-HPLC analysis of the constituents from *G. sylvestre*. It was observed that the chromatogram of 9 components incubated with α-glucosidase in *G. sylvestre* showed higher than the inactivated control group, which indicates these 9 constituents exhibited specific binding toward α-glucosidase and thus were considered as major potential α-glucosidase ligands. Notably, it is the first time to clearly describe the major α-glucosidase ligands in *G. sylvestre*.

Based on the variations of the peak areas before and after incubation with α-glucosidase, the enrichment factors can be used to determined the degree of affinity between the ligand and the enzyme. The enrichment factor (EF) was calculated as follows:

(2) $$\begin{array}{l} \text{EF }\left(\%\right)=\frac{\left(\text{At }-\text{ Ac}\right)}{\text{A}0}\times 100\% \end{array}$$

where At, Ac, and A0 represent the peak areas obtained from incubation of the *G. sylvestre* with activated, inactivated and without α-glucosidase in Figure 2 (Zhu et al., 2013), respectively. Among those chemical constituents from *G. sylvestre*, the unique EF could evaluate the binding affinity between each compound and α-glucosidase, and also suggest the potential hypoglycemic activity. The results showed that compound 9 possessed the greatest degree of affinity (18.92%), followed by compounds 4 (10.35%), 3 (9.24%), 1 (8.62%), 5 (8.16%), and 7 (5.79%) in Table 1. As expected, the EFs for each component were different from each other. Theoretically, the discrepant EFs may be attributed to their competitively distinguished interactions with α-glucosidase.

**Table 1:** **The enrichment factors (EF) and the UF-HPLC-MS data of potential inhibitors of α-glucosidase from the ***G. sylvestre*****.

**No**.	**t_R_(min)**	**EF(%)**	**[M-H]^−^**	**MS/MS data**	**Identification**
1	14.4	8.62	187	187, 169, 125	3,5-Dihydroxyl-6-hydroxymethyl-1-hydroxymethyl phenol ester
2	17.7	4.32	664	664, 542, 487, 175, 113, 87	Gymnemic acid A
3	19.1	9.24	331	331, 329, 313, 201, 157, 127	8-Hydroxy gymnamine
4	19.7	10.35	329	329, 229, 211, 171	9, 10, 13-Trihydroxy octadecenoic acid
5	20.2	8.16	301	301, 283, 265, 221	Hypolaetin
6	20.7	1.52	287	287, 285, 269, 241	Aromadendrin
7	21.8	5.79	503	503, 485, 455, 437	Madecassic acid
8	22.5	3.41	796	796, 525, 407	Alternoside XVIII
9	25.1	18.92	530	485, 454, 410, 208	Unknown

### Structural identification of the α-glucosidase ligands

**Figure 3:**
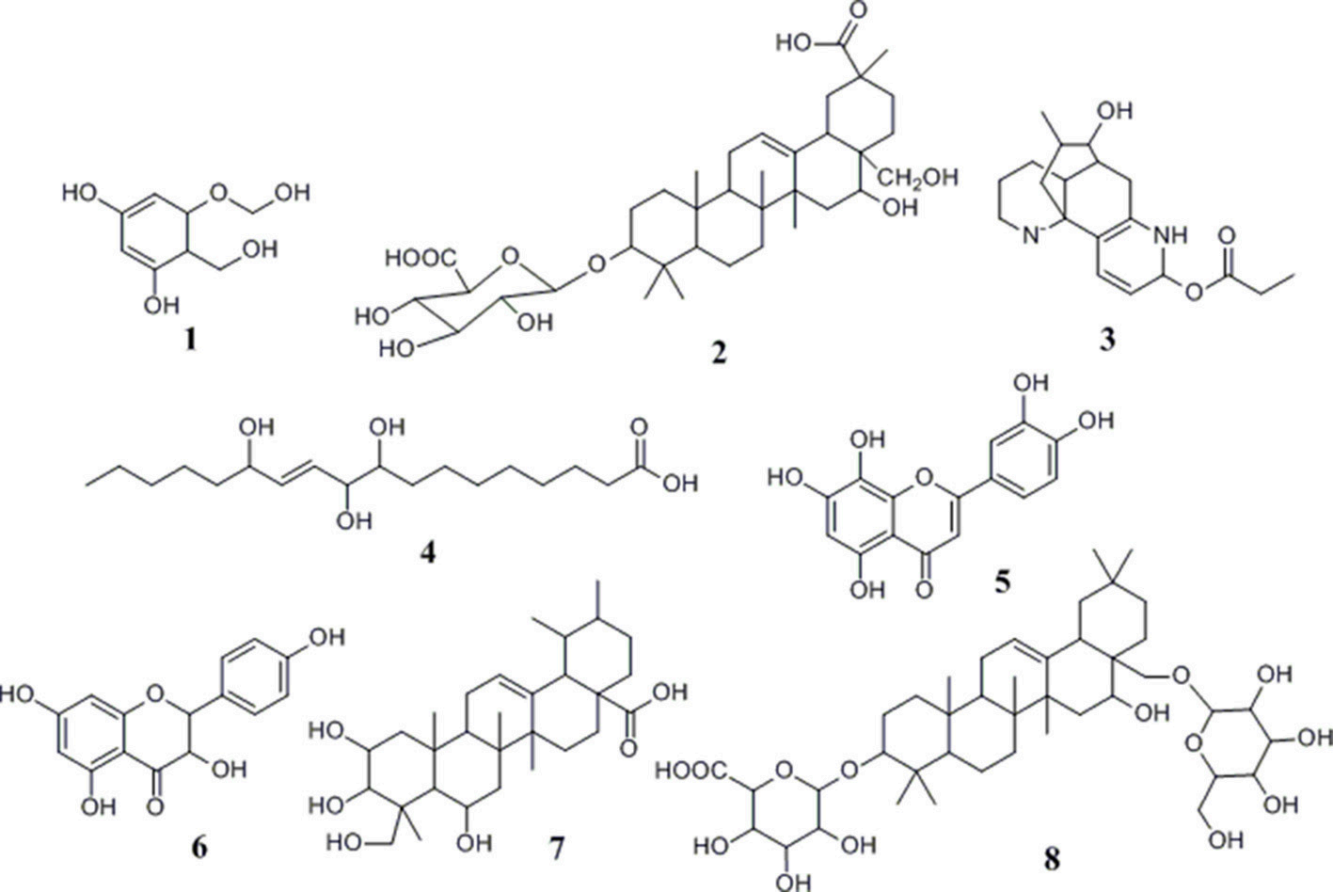
**Chemical structures of compounds identified from ***G. sylvestre*** based on the MS/MS spectra**.

After incubation with α-glucosidase and ultrafiltration affinity screening, the 9 compounds in *G. sylvestre* with different affinities to α-glucosidase were identified by both their HPLC retention time and MS/MS spectra in the negative ion mode. The retention times (t_R_), molecular masses, contents, and fragment ions of the components were listed in Table 1, and their corresponding structures are shown in Figure 3.

**Figure 4:**
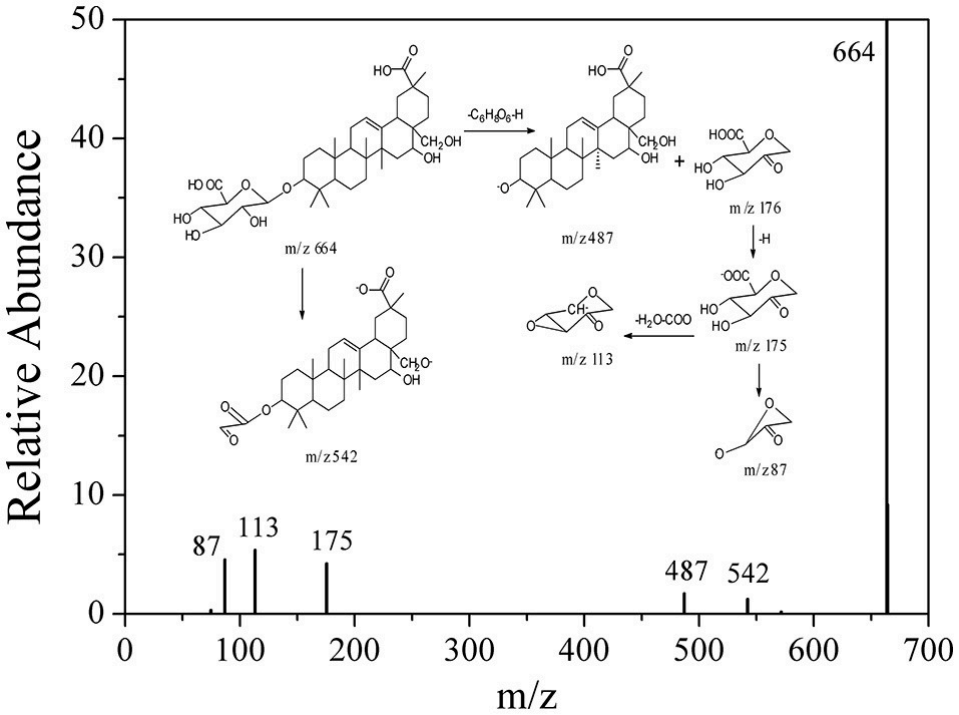
**The fragmentation pathways proposed according to the MS/MS spectra of compound 2**.

Based on these data, the corresponding MS/MS data, fragmentation pathways of some representative compounds have been proposed. By comparing the MS/MS data with those of the previous literatures, compound 3, 4, 5, and 6 were tentatively characterized as 8-hydroxy gymnamine (Rao et al., 1972), 9,10,13-trihydroxy octadecenoic acid (Llorent-Martínez et al., 2015), hypolaetin (Stanoeva and Stefova, 2012), aromadendrin (Yang et al., 2003). Compound 1 presented the [M-H]^−^ ion at *m/z* 187 and further suffered neutral losses of 18 Da (H_2_O) and 44 Da (CO_2_) to produce the fragment ions of the [M-H-H_2_O]^−^ ion at *m/z* 169 and the [M-H-H_2_O-CO_2_]^−^ ion at *m/z* 125. In consideration of the flavonoids characteristic fragment ion of *m/z* 125 generated from the retro-Diels-Alder (RDA) cleavage of C ring, compound 1 was conjectured to be 3,5-dihydroxyl-6-hydroxymethyl-1-hydroxymethyl phenol ester. Compound 2, 7, and 8 shared the same basic skeleton of oleanane type triterpene and were determined to be gymnemic acid A, madecassic acid (Xia et al., 2015) and alternoside XVIII (Yoshikawa et al., 1999). In order to further elucidate the structures of those triterpenoids, the MS/MS spectra and representative fragmentation pathways of compound 2 were shown in Figure 4. Compound 2 was tentatively identified as gymnemic acid A (calculated as C_36_H_56_O_11_, with Mw. 664, Figure 4), which was indicated by a deprotonated aglycone molecular ion [M-GluA-H]^−^ at *m/z* 487 in the negative ion mode due to the neutral loss of glucuronic acid (C_6_H_8_O_6_, 176 Da). Besides, other fragmented ions at *m/z* 542, 175, 113, and 87 were obtained due to the successive dissociation of GluA moiety, since saponins with a four-member ring remain stable and only produce pieces of sugar under the large cracking pressure (Zhao et al., 2017). By comparison of the MS/MS spectra with those of previous study, compound 2 was suggested as gymnemic acid A (Wang et al., 2004).

## Discussion

### Screening and identification for α-glucosidase ligands in *G. sylvestre*

The traditional approaches to screen ligands of an enzyme usually involved repeated chromatographic separations of pure compounds, then followed by bioactivity assays with the corresponding enzyme, which is both labor-intensive, time and material consuming. Fortunately, with the development of the bio-affinity methods based on the interactions between the ligands and the active sites of enzymes, the combination of bio-affinity methods (such as UF) with HPLC-MS techniques could further offer the vital insights into the biomolecular structures and their ligand-receptor binding properties (Zhang et al., 2004; Zhou et al., 2011; Qin et al., 2015). If one ingredient in the mixture is able to interact with a specific enzyme, such as α-glucosidase, the peak area of the bound component will significantly increase in the total ion chromatogram after separated from the enzyme. In this way, UF-HPLC-MS assay could rapidly screen and identify the ligand-receptor complexes from unbound compounds by directly comparing the chromatogram peak areas between activated and inactivated α-glucosidase after ultrafiltration.

As a traditional medicinal plant in Asia for thousands of years, *G. sylvestre* has been widely used as a remedy for the diabetes mellitus. It has been reported that the extract of *G. sylvestre* could decrease the taste sensitivity to sweetness, glucose absorption in the gastrointestinal tract and increase plasma insulin level by repairing or regenerating the pancreatic islet (Izutani et al., 2005; Ahmed et al., 2010). This folk medicine also possesses the properties for the treatment of snake bite, eye complaints, stomachic and diuretic problems, and asthma (Praveen et al., 2014). For the further chemical and pharmacological researches, saponin 21β-*O*-benzoylsitakisogenin 3-*O*-β-D-glucopyranosyl (1 → 3)-β-D-glucuronopyranoside and the sodium salt of alternoside II showed sweetness-inhibiting activity in adult volunteers (Ye et al., 2001), and gymnemoside b, gymnemic acids III, V, and VII exhibited weak inhibitory activity on plasma glucose in rats during the oral glucose tolerance test (Yoshikawa et al., 1997). In this study, *G. sylvestre* showed remarkable inhibitory effect on α-glucosidase with IC_50_ at 68.70 ± 1.22 μg/mL, compared with the positive control acarbose at 59.03 ± 2.30 μg/mL. The efficacy of natural products usually depended on the characteristic of its chemical ingredients. In order to further clarify and analyze the active components in *G. sylvestre*, the UF-HPLC-MS analysis with α-glucosidase was developed and detailed afterwards.

The result of UF-HPLC-MS analysis suggested that 9 components in *G. sylvestre* exhibited specific binding toward α-glucosidase. With the highest bio-affinity of the compound 4, the 9 compounds with discrepant EFs disclosed competitively distinguished interactions with α-glucosidase. To this end, these 9 compounds were further identified by both their HPLC retention times (t_R_) and MS/MS spectra.

### The structure-activity relationships between ligands and α-glucosidase

Up to now, more than 30,000 terpenes have been isolated from this plant. Due to their relatively complex structures, triterpenoids in free form (sapogenins), linked to glycosides (saponins) or acetylated are vital and widely exist in plants, which have exerted important pharmacological functions both *in vitro* and *in vivo* researches (Fabio et al., 2014). Generally speaking, structural diversity of triterpenoids based on substituent groups in *G. sylvestre* could largely contribute to their diversified biological activities. Commonly, the acyl groups of these triterpenoids might be an important role in the antisweet activity. For example, gymnemic acids III, IV, and VIII-X could inhibit the sweet taste completely at the 0.2 M sucrose (Yoshikawa et al., 1991, 1992). Furthermore, the reduced number of acyl groups of these triterpenoids could lead to the decrease of the antisweet activity. An aliquot of 0.5 mM sample solution of gymnemic acid I, II, XI and XII inhibited the perception of sweetness at the dose of 0.4 M sucrose, which also suggested that the number of acyl groups positively correlated to the anti-sweet activity of these triterpenes (Yoshikawa et al., 1992). Our data summarized in Table 1 and Figure 3 showed that triterpenoid sapogenin (compound 7) exhibited higher EFs than the saponins (compound 2 and 8), which suggested that glycosylation could decrease the potential antisweet activity of sapogenins to some extent. Besides, those results in Table 1 also suggested that other components in *G. sylvestre*, such as flavonoids and alkaloids, could also exert good affinity activity to α-glucosidase. Their higher EFs may due to the high mass spectral responsivity than triterpenoids. Finally, there also existed the synergistic antisweet effects among all those components mentioned above. In some ways, this study could contribute to better knowledge of the hypoglycemic mechanism of *G. sylvestre*.

## Conclusion

In the present work, a simple and rapid method using UF-HPLC-MS has been developed for screening and identifying the α-glucosidase inhibitors from *G. sylvestre*. The extract of this plant exhibited significant inhibitory activity against α-glucosidase with IC_50_ at 68.70 ± 1.22 μg/mL compared with the acarbose at 59.03 ± 2.30 μg/mL. The UF-HPLC-MS assay revealed that a total of 9 components were considered as potential α-glucosidase inhibitors, and their chemical structures were characterized and identified subsequently. Meanwhile, the structure-activity relationships revealed that glycosylation could decrease the potential antisweet activity of sapogenins. Futhermore, other components in *G. sylvestre*, such as flavonoids and alkaloids, could also be good α-glucosidase inhibitors due to their synergistic effects. To our best knowledge, the UF-HPLC-MS method was, for the first time, applied to comprehensively screen and identify the major α-glucosidase ligands in *G. sylvestre*. In conclusion, the obtained results indicate that the UF-HPLC-MS method could be an effective technique to rapidly screen and identify bioactive ingredients from the complex natural products in order to discovery better natural inhibitors against α-glucosidase and more potential natural anti-diabete candidates.

## Author contributions

MG conceived, designed, and supervised the study; GC performed the experiments, analyzed the data, and wrote the manuscript. All authors approved and reviewed the final manuscript.

### Conflict of interest statement

The authors declare that the research was conducted in the absence of any commercial or financial relationships that could be construed as a potential conflict of interest.
